# Supplementary material for: Gut microbiome signatures of Yorkshire Terrier enteropathy during disease and remission
Source: Sci Rep. 2023 Mar 16;13:4337. doi: 10.1038/s41598-023-31024-w (PMC10018597; doi:10.1038/s41598-023-31024-w)

Supplementary Material

Fig. S1. Principal Component Analysis of clinical and clinicopathological parameters does not segregate the three studied groups (YTE, Remission and Control). The test included as parameters were complete blood count (CBC), serum biochemical profile, measurement of bile acids and basal cortisol concentrations, serum concentration of cTLI (canine trypsin-like- immunereactivity), SpecPL (specific pancreatic lipase), and cobalamin. Additionally, urinalysis for urine protein creatinine ratio (UPC) and urinary sediments.

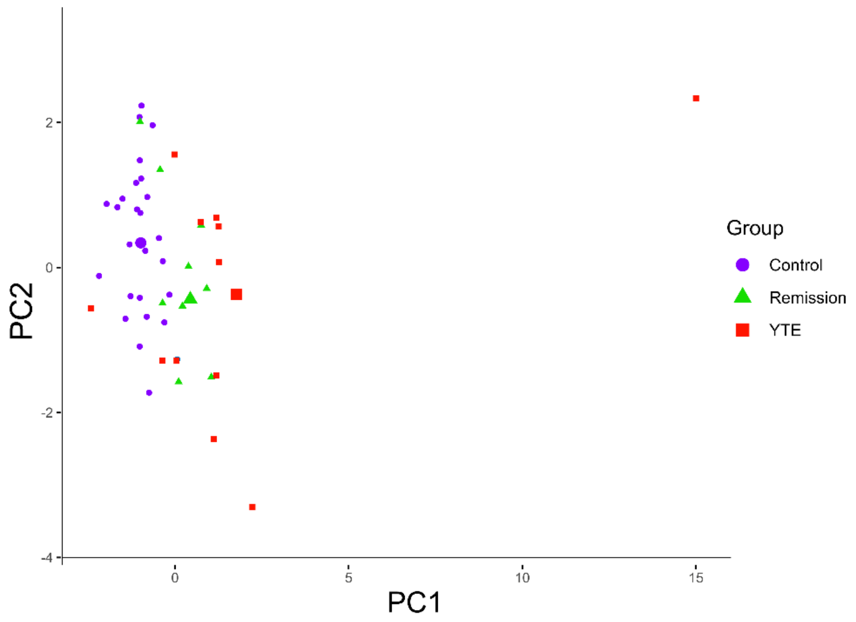

Supplement: Supplementary file 1 — Supplementary Information. [file 41598_2023_31024_MOESM1_ESM.pdf]
