# Supplementary material for: Gut microbiome signatures of Yorkshire Terrier enteropathy during disease and remission
Source: Sci Rep. 2023 Mar 16;13:4337. doi: 10.1038/s41598-023-31024-w (PMC10018597; doi:10.1038/s41598-023-31024-w)
Supplement: Supplementary file 2 — Supplementary Information. [file 41598_2023_31024_MOESM2_ESM.pdf]

Fig. S2. Analysis of the differentially abundant fraction expressed as numbers of reads of 16S rRNA gene for individual dogs and the taxonomic breakdown of the dominant ASVs across all samples.

JMF-2101-12 | gene: 16S rRNA V4 | state: refined | filter min count: 5000 | agglomerated by rank: Domain

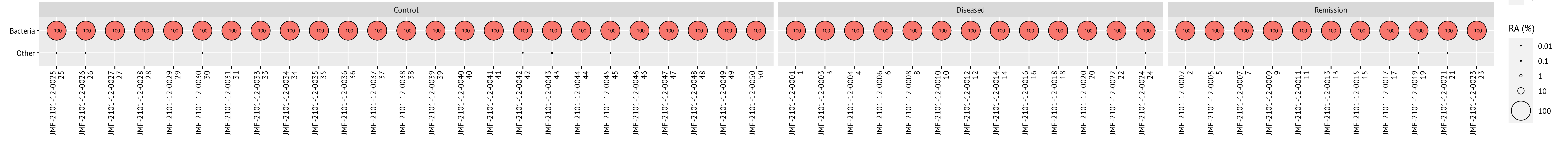

Prevalence collapse settings: "Others" except ≥1% RA in at least 3 samples.

JMF-2101-12 | gene: 16S rRNA V4 | state: refined | filter min count: 5000 | agglomerated by rank: Phylum

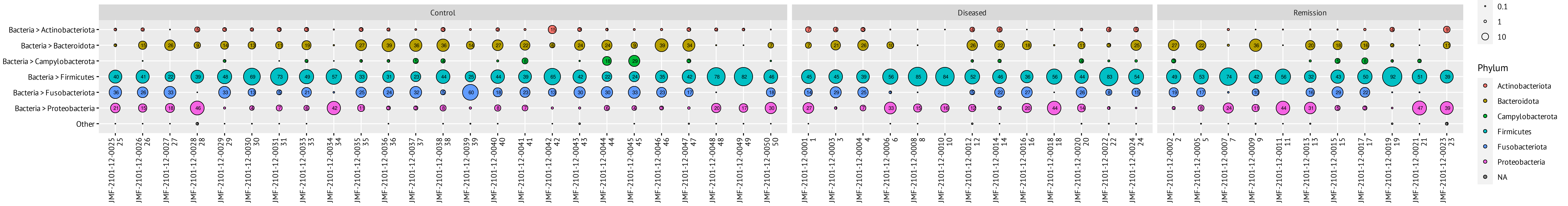

Prevalence collapse settings: "Others" except ≥1% RA in at least 3 samples.

JMF-2101-12 | gene: 16S rRNA V4 | state: refined | filter min count: 5000 | agglomerated by rank: Class

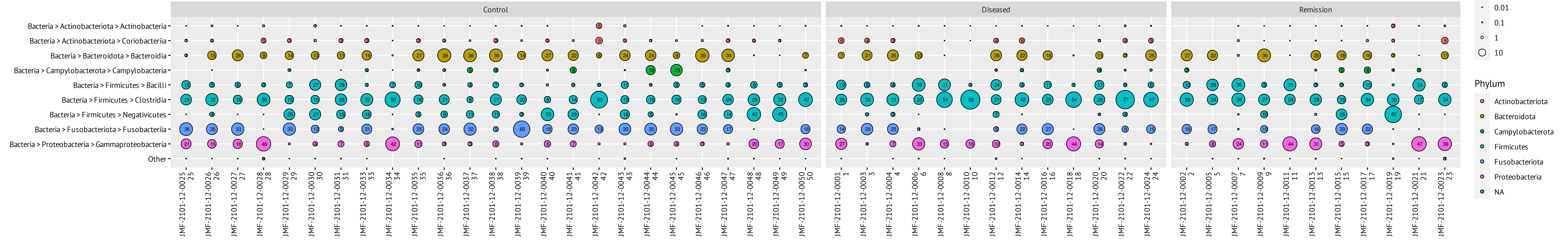

Prevalence collapse settings: "Others" except ≥1% RA in at least 3 samples.

JMF-2101-12 | gene: 16S rRNA V4 | state: refined | filter min count: 5000 | agglomerated by rank: Order

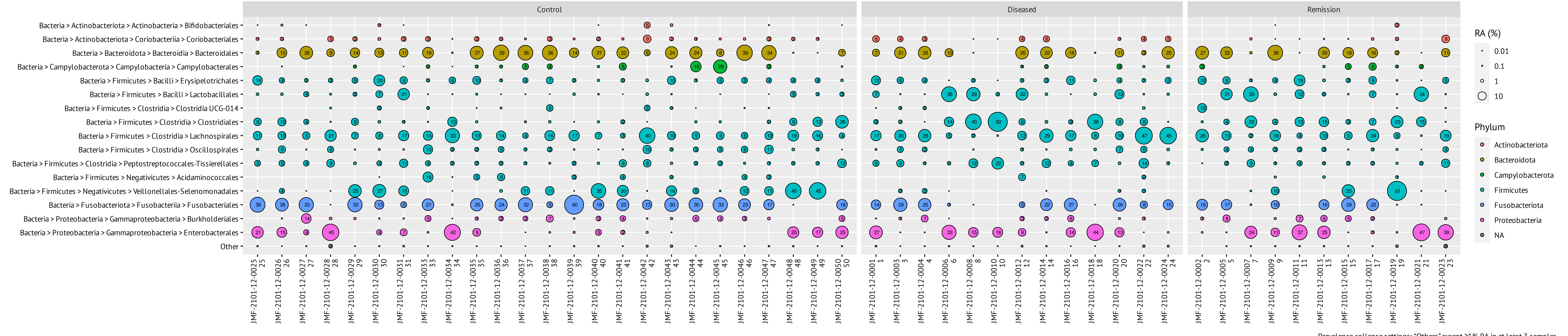

Prevalence collapse settings: "Others" except ≥1% RA in at least 3 samples.

JMF-2101-12 | gene: 16S rRNA V4 | state: refined | filter min count: 5000 | agglomerated by rank: Family

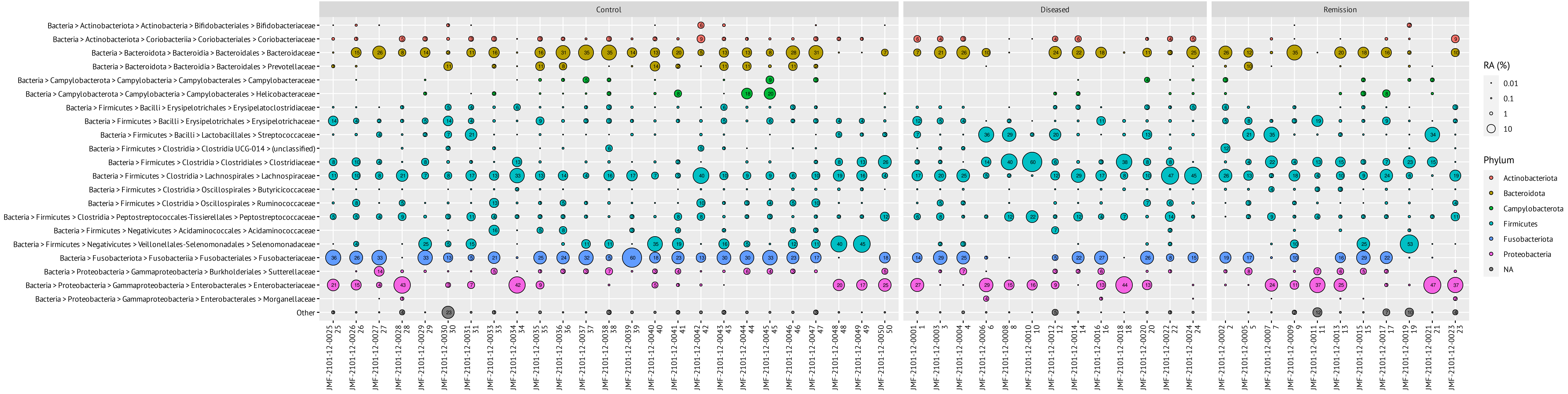

Prevalence collapse settings: "Others" except ≥1% RA in at least 3 samples.

JMF-2101-12 | gene: 16S rRNA V4 | state: refined | filter min count: 5000 | agglomerated by rank: Genus

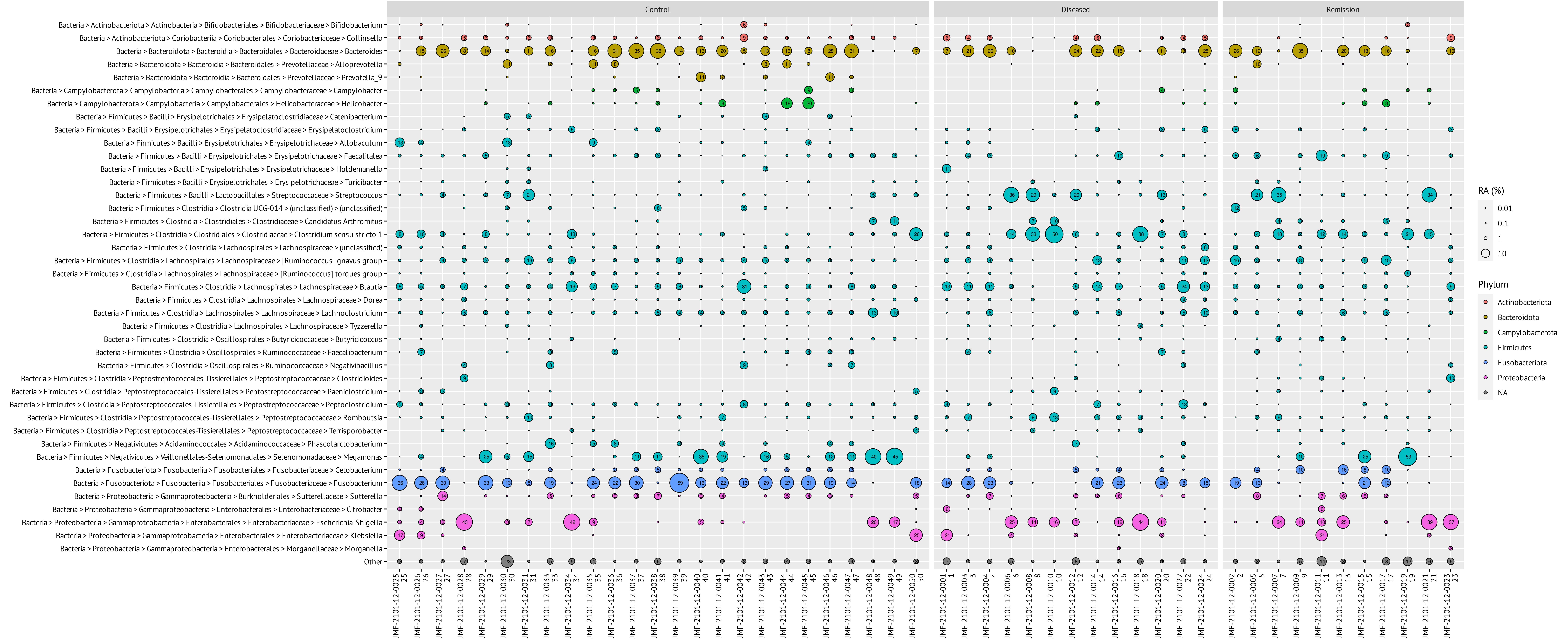

Prevalence collapse settings: "Others" except ≥1% RA in at least 3 samples.

JMF-2101-12 | gene: 16S rRNA V4 | state: refined | filter min count: 5000 | agglomerated by rank: Species

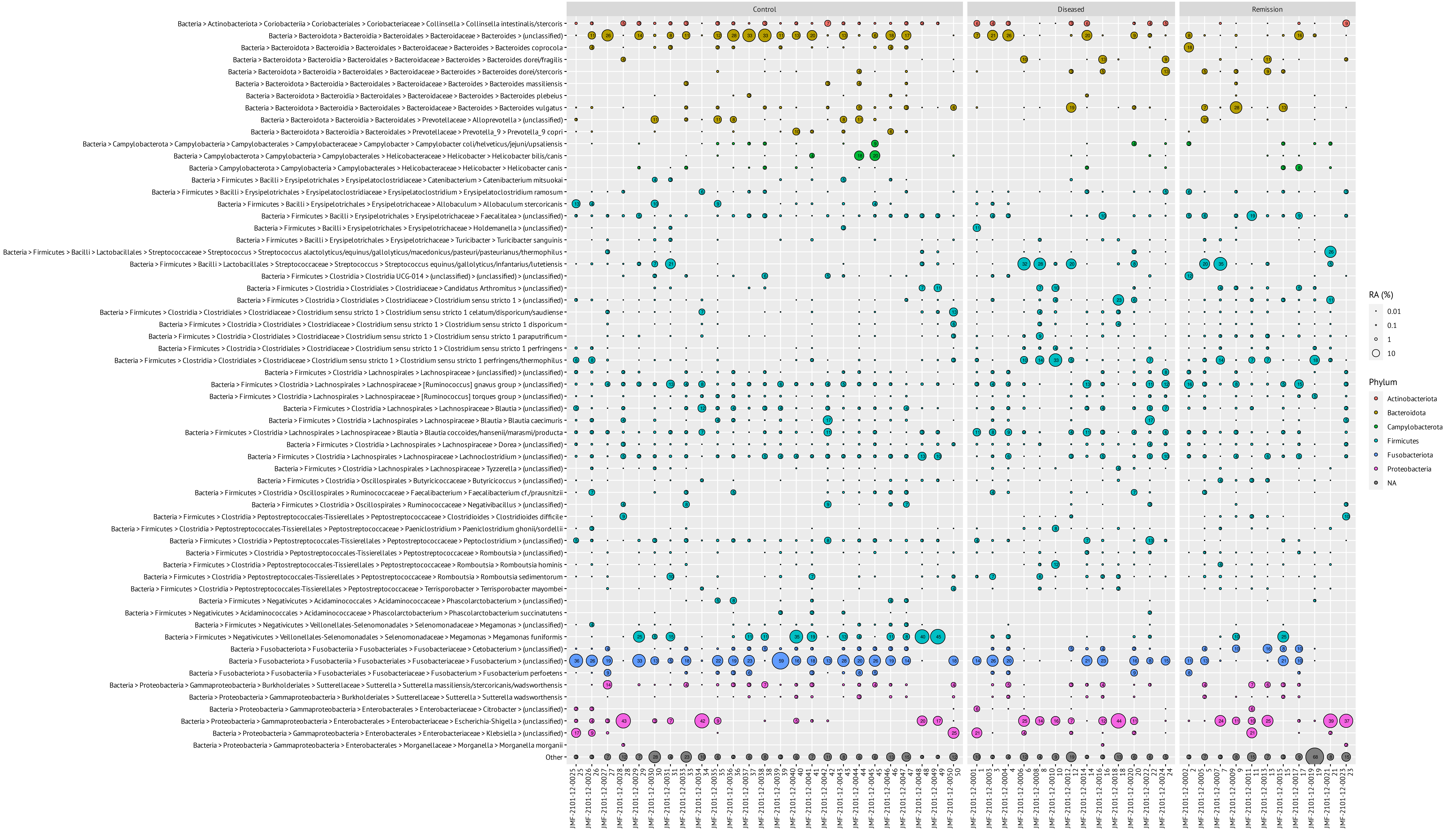

Prevalence collapse settings: "Others" except ≥1% RA in at least 3 samples.

JMF-2101-12 | gene: 16S rRNA V4 | state: refined | filter min count: 5000

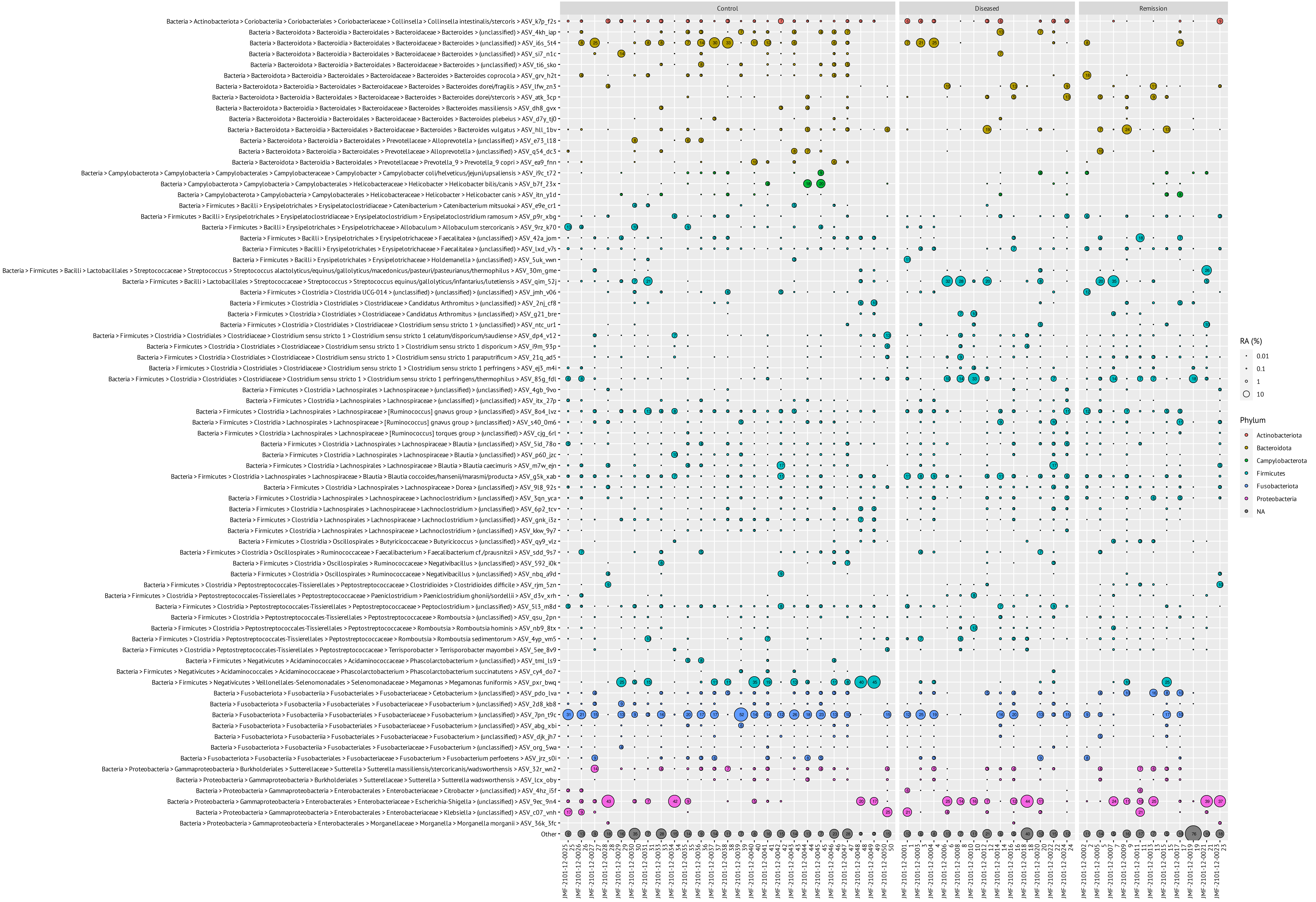

Prevalence collapse settings: "Others" except ≥1% RA in at least 3 samples.
